# Construction and validation of a prediction model for graft failure in pediatric liver transplant recipients based on the APRI-ALBI score: a retrospective study of 289 cases

**DOI:** 10.1080/07853890.2026.2654909

**Published:** 2026-05-08

**Authors:** Quanhai Zhang, Haifeng Tang, Xiaoke Dai, Mingman Zhang

**Affiliations:** Department of Hepatobiliary Surgery, Children’s Hospital of Chongqing Medical University, National Clinical Research Center for Children and Adolescents’ Health and Diseases, Ministry of Education Key Laboratory of Child Development and Disorders, Chongqing, China

**Keywords:** Apri-ALBI, pediatric liver transplantation, graft failure

## Abstract

**Objective:**

• • •

**Background:**

To develop and validate a preoperative APRI-ALBI-based nomogram for predicting graft failure (GF) after pediatric liver transplantation.

**Methods:**

A single-center, retrospective cohort of 289 children who underwent primary liver transplantation for end-stage liver disease between April 2017 and September 2024 was enrolled. Forty-two recipient, donor and intra-operative variables were collected. Candidate predictors were first screened by univariate Logistic/Cox analysis, further reduced by LASSO regression with 10-fold cross-validation, and finally entered into multivariable models. A nomogram integrating APRI-ALBI and other independent risk factors was constructed. Model discrimination, calibration and clinical utility were assessed with ROC analysis, calibration plots, Hosmer-Lemeshow test, decision-curve analysis (DCA) and restricted cubic splines (RCS). Kaplan–Meier curves and time-dependent ROC were used to validate 1- and 3-year GF prediction.

**Results:**

Multivariable Logistic/Cox analysis identified six independent predictors of GF: MELD score, male donor, APRI-ALBI score, donor height, operative time and intra-operative blood loss. The resulting nomogram achieved an AUC of 0.706, outperforming PELD, MELD and Child-Pugh scores, with good calibration (Hosmer-Lemeshow *p* > 0.05) and favorable net benefit on DCA. RCS revealed a dose-dependent increase in GF risk once APRI-ALBI exceeded 10. Kaplan–Meier analysis showed significantly lower GF-free survival in the high APRI-ALBI group (*p* < 0.01). Time-dependent ROC curves demonstrated excellent predictive accuracy at 30 days and 1 year (AUC 0.899–0.977).

**Conclusion:**

APRI-ALBI is an independent risk factor for GF in pediatric liver transplantation. The APRI-ALBI-based nomogram offers robust discrimination, calibration and clinical utility for individual risk stratification.

## Introduction

End-stage liver disease (ESLD) is one of the leading indications for pediatric liver transplantation (PLT) [1,2]. Although surgical techniques, peri-operative management and immunosuppressive protocols have improved continuously, graft failure (GF) remains the predominant cause of early graft loss and death within 90 days after transplantation, with an incidence of 4–12% and a near 100% mortality once it occurs [3,4]. The pathogenesis of GF is complex, encompassing primary non-function (PNF), early acute rejection (EAR), small-for-size syndrome (SFSS) and ischaemia–reperfusion injury coupled with microvascular biliary damage [5–7]. In children, the clinical manifestations are often atypical, making early recognition difficult and delaying optimal intervention.

Currently available risk-stratification tools for GF after liver transplantation – such as MELD, PELD and Child–Pugh scores – were derived from adult cohorts and exhibit limited applicability and suboptimal predictive performance in the paediatric population [8,9]. Moreover, traditional models frequently rely on subjective indices (e.g. hepatic encephalopathy grade) or expensive dynamic tests such as indocyanine green clearance (ICG), which are impractical in resource-limited paediatric centres [10]. An inexpensive, objective model based on routinely available laboratory parameters is therefore urgently needed to enable early identification and precise management of GF risk.

Simple serological scores have recently gained attention. The aspartate aminotransferase-to-platelet ratio index (APRI) and the albumin–bilirubin (ALBI) score are reproducible, easy to calculate and have shown promise in assessing hepatic fibrosis, functional reserve and post-operative complications [11,12]. In adults, the combined APRI–ALBI score predicts post-hepatectomy liver failure and 30-day mortality with AUCs of 0.70–0.766 [13–15]. However, its value for predicting GF in children undergoing liver transplantation remains unexplored.

Traditional Cox/logistic models are prone to overfitting caused by multicollinearity. Least absolute shrinkage and selection operator (LASSO) regression, which penalises regression coefficients to perform high-dimensional variable selection, has been increasingly applied in transplantation research [16–18]. We hypothesised that integrating APRI–ALBI into multivariable LASSO-logistic and LASSO-Cox frameworks would mitigate overfitting, enhance predictive accuracy and yield a visualisable nomogram for individual risk stratification.

This single-centre retrospective study of 289 paediatric liver-transplant recipients aimed to: (i) delineate the independent association between APRI–ALBI and GF; (ii) identify key predictors *via* LASSO-logistic regression and construct an internally validated nomogram; and (iii) quantify the impact of APRI–ALBI on GF-free survival using a LASSO-Cox model that treats GF as a competing risk, with discrimination and clinical net benefit assessed by time-dependent ROC and decision-curve analyses.

## Patients and methods

This retrospective cohort study was conducted in accordance with the Declaration of Helsinki and was approved by the Ethics Committee of the Children’s Hospital Affiliated to Chongqing Medical University [Approval No. (2025) Lun Shen (Lin Yan) No. (78)]. The requirement for informed consent was waived due to the retrospective nature of the study. We hereby declare that none of the organs/tissues were procured from executed prisoners. All organs were procured after informed consent was obtained from the donors or their legal representatives.

## Patients

Children with end-stage liver disease who underwent their first liver transplantation at the Department of Hepatobiliary Surgery, Children’s Hospital of Chongqing Medical University, between April 2017 and September 2024 were considered for inclusion.

**Inclusion criteria:**

1. age < 18 years;
2. diagnosis of end-stage liver disease undergoing first liver transplantation;
3. complete pre-operative clinical data and post-operative follow-up information available.

End-stage liver disease (ESLD) was defined according to the 2014 AASLD Practice Guidelines for the evaluation of pediatric liver transplant candidates [19,20]. Patients were considered to have ESLD if they presented with: (i) chronic cholestatic or cirrhotic disease with a Pediatric End-Stage Liver Disease (PELD) score or Model for End-Stage Liver Disease (MELD) score necessitating transplantation; (ii) acute liver failure, defined by severe synthetic dysfunction (INR ≥ 2.0) without or with hepatic encephalopathy; (iii) metabolic or genetic conditions leading to progressive hepatic decompensation; (iv) manifestations of clinical decompensation, including failure to thrive (significant growth retardation), refractory ascites, variceal bleeding, or hepatopulmonary syndrome. All patients in the study cohort were evaluated by a multidisciplinary team and met the standardized criteria for primary liver transplantation.

**Exclusion criteria:**

1. multi-organ transplantation (e.g. combined liver–kidney);
2. pre-operative malignant tumours;
3. missing key clinical or laboratory variables.Specifically, patients were excluded if they lacked essential baseline laboratory data (pre-transplant albumin or INR) required to calculate the APRI-ALBI score, or if they were lost to follow-up within the first year post-transplantation, preventing the accurate determination of graft survival status.

## Definitions

No universal definition for graft failure (GF) exists; however, according to the European Association for the Study of the Liver (EASL) guidelines GF is generally recognised as irreversible graft injury due to primary non-function, early allograft dysfunction, acute or chronic rejection, disease recurrence, or immune-mediated damage, ultimately requiring re-transplantation or resulting in patient death [21].

### Data collection

All variables were retrospectively extracted from the hospital electronic medical record system and comprised laboratory values obtained within 7 days before transplantation, recipient and donor characteristics, and intra-operative parameters.

In this study, Warm Ischemia Time (WIT) was defined specifically as the first warm ischemia time (donor procurement WIT), representing the interval from the cessation of donor hepatic circulation (cross-clamping) to the initiation of cold flush preservation [22].

The primary endpoint of this study was early graft failure, which was strictly defined as graft loss (resulting in patient death or requiring re-transplantation) occurring within 90 days following the initial liver transplantation.

Calculation of ALBI, APRI, Child-Pugh, PELD, MELD and APRI–ALBI scores
APRI = [AST (U/L)/upper limit of normal AST (U/L)] × 100/platelet count (×10^9^/L) [23].
ALBI = 0.66 × log_10_ (total bilirubin, µmol/L) – 0.085 × albumin (g/L) [24].
APRI–ALBI = 5.280 × ALBI + 1.583 × APRI [25].

Child-Pugh class was determined as reported previously and patients were categorised as class A (5–6 points), B (7–9 points) or C (10–15 points) [26].

PELD = 4.80 × ln(total bilirubin, mg/dL) + 6.87 × ln(INR) + 4.37 × ln(albumin, g/dL) [27].
MELD = 3.78 × ln(total bilirubin, mg/dL) + 11.2 × ln(INR) + 9.57 × ln(creatinine, mg/dL) + 6.43 [28].

### Statistical analysis

Continuous variables are presented as median (inter-quartile range, IQR) and were compared with the Mann–Whitney U test. Categorical data are expressed as frequency (percentage) and were analysed with the χ^2^ or Fisher exact test. Variables significant in univariate logistic or Cox regression were advanced to subsequent analyses. To minimise multicollinearity and select key predictors, LASSO regression was applied before multivariable logistic and Cox modelling. The final logistic model was visualised with a nomogram. Discrimination was quantified with receiver-operating-characteristic (ROC) curves and area under the curve (AUC); AUCs were compared with DeLong test. Model calibration was assessed with the Hosmer–Lemeshow goodness-of-fit test, and clinical net benefit across risk thresholds was evaluated with decision-curve analysis (DCA). For the final Cox model, Kaplan–Meier curves were constructed and compared with the log-rank test. Time-dependent AUCs at 30 days, 90 days and 1 year were calculated. Bootstrap calibration (*B* = 500) was used to internally validate both the logistic and Cox models. Restricted cubic splines were employed to examine dose–response relationships. A two-sided *p* < 0.05 was considered statistically significant. All analyses were performed with R software version 4.5.1.

## Results

A total of 289 children who underwent liver transplantation were retrospectively reviewed. Seventeen (5.9%) developed GF and 272 (94.1%) did not. Baseline comparisons (Table 1) showed that children with GF were older (8.37 vs 5.90 months, *p* = 0.023), had lower pre-operative albumin (32.30 vs 38.15 g/L, *p* = 0.001), lower platelet and lymphocyte counts (both *p* < 0.05), and a significantly higher APRI–ALBI score (–0.90 vs −3.87, *p* = 0.004). Donor characteristics (Table 2) revealed a higher proportion of male donors in the GF group (76.47% vs 44.12%, *p* = 0.019). Intra-operative parameters (Table 3) demonstrated longer cold ischaemia time, total operative time, anhepatic phase, portal-to-arterial anastomosis time and greater intra-operative blood loss (all *p* < 0.05).

**Table 1.** Baseline characteristics of pediatric patients undergoing liver transplantation.

	Graft failure group (*n* = 17)	Non-graft failure group (*n* = 272)	P-value
**Demographics**			
**Age (months)**	8.37 (6.97, 28.20)	5.90 (5.07, 11.32)	**0.023**
**Gender**			0.802
Male	7 (41.18)	129 (47.43)	
Female	7 (41.18)	129 (47.43)	
BMI (kg/m²)	16.23 (14.86, 17.33)	16.16 (14.89, 17.44)	0.907
**Disease Severity**			
PELD	20.04 (13.07,35.20)	16.15 (7.74,21.27)	0.06
MELD	20.53 (16.61,32.00)	18.64 (15.51,21.92)	0.087
Child-Pugh	8.00 (5.00, 11.00)	9.00 (7.00, 10.00)	0.847
**Primary Diagnosis**			0.1
BA	10 (58.82)	215 (79.04)	
Others	7 (41.18)	57 (20.96)	
**Preoperative Labs**			
PLT (×10⁹/L)	180.00 (84.00, 311.00)	265.50 (183.00, 378.00)	**0.036**
LYM (×10⁹/L)	5.09 (1.31, 8.32)	7.36 (3.70, 10.30)	**0.047**
TB (μmol/L)	304.00 (124.90, 371.60)	231.30 (131.58, 295.85)	0.189
DBIL (μmol/L)	176.20 (59.40, 259.80)	170.60 (76.28, 238.57)	0.52
ALB (g/L)	32.30 (26.20, 36.30)	38.15 (33.95, 43.32)	**0.001**
ALT (U/L)	145.60 (76.60, 270.00)	183.65 (92.15, 286.40)	0.73
AST (U/L)	280.50 (186.50, 496.50)	293.00 (156.85, 416.30)	0.579
Cr (μmol/L)	20.50 (13.40, 23.40)	16.60 (13.82, 22.00)	0.385
PT (s)	13.20 (12.50, 17.30)	13.70 (12.00, 16.50)	0.399
INR	1.18 (1.11, 1.49)	1.20 (1.06, 1.47)	0.567
AP (U/L)	350.00 (232.00, 588.00)	598.50 (382.75, 832.75)	**0.007**
CHE (U/L)	3291.00 (2322.00, 4260.00)	3823.50 (2737.50, 5440.00)	0.171
GGT (U/L)	119.50 (54.00, 264.00)	324.75 (125.50, 718.25)	**0.031**
APRI-ALBI	−0.90 (−2.66, 4.13)	−3.87 (−8.24, −0.67)	**0.004**

**PLT**: Platelets; LYM: Lymphocytes; **ALT**: Alanine Aminotransferase; **AST**: Aspartate Aminotransferase; **ALP**: Alkaline Phosphatase; **GGT**: Gamma-Glutamyl Transferase; **TB**: Total Bilirubin; **DBIL**: Direct Bilirubin; **ALB**: Albumin; **CHE**: Cholinesterase; **Cr**: Creatinine; **PT**: Prothrombin Time; **INR**: International Normalized Ratio; **APRI**: AST to Platelet Ratio Index; **ALBI**: Albumin-Bilirubin; **PELD**: Pediatric End-Stage Liver Disease; **MELD**: Model for End-Stage Liver Disease.

**Notes**: Data presented as median (IQR) or n (%). Other etiologies in the GF group (*n* = 7) included cavernous transformation of the portal vein (*n* = 1), cirrhosis (*n* = 1), Alagille syndrome (*n* = 1), fulminant hepatic failure (*n* = 1), hepatic hemangioma (*n* = 1), primary biliary cholangitis (*n* = 1), and overlap syndrome (*n* = 1). Other etiologies in the non-GF group (*n* = 56) included cavernous transformation of the portal vein (*n* = 10), Wilson’s disease (*n* = 8), cirrhosis (*n* = 7), Caroli disease (*n* = 6), urea cycle disorders (*n* = 5), progressive familial intrahepatic cholestasis type 3 (*n* = 4), portal venous flow anomalies (*n* = 5), Alagille syndrome (*n* = 3), fulminant hepatic failure (*n* = 3), glycogen storage disease (*n* = 3), hepatic echinococcosis (*n* = 1), Langerhans cell histiocytosis (*n* = 1), and carbamoyl phosphate synthetase I deficiency (*n* = 1).

**Table 2.** Donor baseline characteristics.

	Graft failure group (*n* = 17)	Non-graft failure group (*n* = 272)	P-value
**Donor Gender**			0.019
Male	13 (76.47)	120 (44.12)	–
Female	4 (23.53)	152 (55.88)	–
**Donor source**			**<0.001**
LDLT	7 (41.11)	231 (84.92)	
DCD	10 (58.82)	41 (15.07)	
**Donor Age (years)**	28.15 (25.33, 37.00)	28.88 (25.56, 32.60)	0.911
**Donor BMI**	22.86 (20.28, 23.88)	22.74 (20.34, 24.95)	0.649

**LDLT**: living donor liver transplantation; **DCD**: donation after circulatory death.

**Notes**: Data presented as median (IQR) or n (%).

**Table 3.** Intraoperative parameters.

	Graft failure group (*n* = 17)	Non-graft failure group (*n* = 272)	P-value
Warm Ischemia Time (min)	2.00 (0.83, 4.70)	1.50 (1.08, 2.25)	0.653
Cold Ischemia Time (min)	240.00 (112.00, 440.00)	87.00 (72.75, 136.25)	<0.001
Total Operation Time (h)	10.17 (7.25, 12.17)	7.62 (6.83, 8.55)	0.003
Anhepatic Phase (min)	76.00 (58.00, 95.00)	51.00 (44.00, 63.00)	<0.001
Portal Perfusion to Arterial Anastomosis Time (min)	87.00 (70.00, 118.00)	70.00 (59.75, 86.00)	0.015
Blood Loss	1200.00 (850.00, 1900.00)	350.00 (250.00, 500.00)	<0.001
GRWR	3.22 (2.67, 4.77)	3.65 (2.82, 4.34)	0.905

**GRWR**: Graft-to-Recipient Weight Ratio.

**Notes**: Data presented as median (IQR) or n (%).

### Multivariable logistic model for paediatric GF (LASSO-selected)

Univariate logistic analysis identified PELD (OR = 1.033, *p* = 0.024), MELD (OR = 1.063, *p* = 0.012), platelet count (OR = 0.996, *p* = 0.038), albumin (OR = 0.880, *p* = 0.002), alkaline phosphatase (OR = 0.998, *p* = 0.013) and APRI–ALBI (OR = 1.170, *p* = 0.003) as significant. After LASSO screening, multivariable analysis retained MELD (OR = 1.065, *p* = 0.046) as an independent predictor (Table 4).

**Table 4.** Univariate and multivariate logistic regression analysis of pediatric patients undergoing liver transplantation.

Variable	Univariate OR (95% CI)	P-value	Multivariate OR (95% CI)	P-value
**Recipient Baseline Characteristics**				
	1.007 (0.997–1.016)	0.112	–	**–**
Gender	1.289 (0.481–3.642)	0.617	–	–
Height (cm)	1.011 (0.994–1.026)	0.163	–	–
Weight (kg)	1.036 (0.990–1.077)	0.086	–	–
Recipient BMI (kg/m²)	1.019 (0.837–1.169)	0.826	–	–
**Disease Severity**				
PELD	1.033 (1.003–1.062)	**0.024**	–	–
MELD	1.063 (1.011–1.114)	**0.012**	1.065 (0.999–1.134)	0.046
Child-Pugh	0.993 (0.867–1.153)	0.919	–	–
**Preoperative Labs**				
PLT (×10⁹/L)	0.996 (0.992–1.000)	**0.038**	–	–
Hb (g/L)	1.005 (0.995–1.013)	0.203	–	–
LYM (×10⁹/L)	0.886 (0.772–0.999)	0.063	–	–
TB (μmol/L)	1.002 (0.999–1.006)	0.242	–	–
DBIL (μmol/L)	1.002 (0.997–1.006)	0.424	–	–
ALB (g/L)	0.880 (0.809–0.950)	**0.002**	–	–
ALT (U/L)	1.000 (0.996–1.002)	0.802	–	–
AST (U/L)	1.001 (0.999–1.003)	0.427	–	–
Cr (μmol/L)	0.999 (NA − 1.007)	0.92	–	–
PT (s)	1.016 (0.939–1.073)	0.629	–	–
INR	1.103 (0.446–2.009)	0.788	–	–
AP (U/L)	0.998 (0.996–0.999)	**0.013**	–	–
CHE (U/L)	1.000 (1.000 − 1.000)	0.246	–	–
GGT (U/L)	0.998 (0.996–1.000)	0.065	–	–
APRI-ALBI	1.170 (1.067–1.314)	**0.003**	1.131 (1.014–1.312)	0.067

**PLT**: Platelets; LYM: Lymphocytes; **ALT**: Alanine Aminotransferase; **AST**: Aspartate Aminotransferase; **ALP**: Alkaline Phosphatase; **GGT**: Gamma-Glutamyl Transferase; **TB**: Total Bilirubin; **DBIL**: Direct Bilirubin; **ALB**: Albumin; **CHE**: Cholinesterase; **Cr**: Creatinine; **PT**: Prothrombin Time; **INR**: International Normalized Ratio; **APRI**: AST to Platelet Ratio Index; **ALBI**: Albumin-Bilirubin; **PELD**: Pediatric End-Stage Liver Disease; **MELD**: Model for End-Stage Liver Disease.

Donor variables (Table 5) showed that male donor sex was significant in both univariate (OR = 0.243, *p* = 0.016) and multivariate (OR = 0.142, *p* = 0.014) logistic models. Among intra-operative factors (Table 6), total operative time remained independently associated with GF (OR = 1.517, *p* = 0.002) after LASSO selection.

**Table 5.** Donor baseline characteristics.

Variable	Univariate OR (95% CI)	P-value	Multivariate OR (95% CI)	P-value
Donor Gender	0.243 (0.067–0.706)	**0.016**	0.142 (0.024–0.582)	**0.014**
Donor Age (years)	0.988 (0.940–1.046)	0.656	–	–
Donor Height (cm)	0.979 (0.964–0.998)	**0.016**	1.013 (0.997–1.027)	0.07
Donor Weight (kg)	0.979 (0.953–1.009)	0.146	–	–
Donor BMI (kg/m²)	0.933 (0.817–1.068)	0.31	–	–

**Table 6.** Intraoperative parameters.

Variable	Univariate OR (95% CI)	P-value	Multivariate OR (95% CI)	P-value
Warm Ischemia Time (min)	1.116 (0.896–1.322)	0.248	–	–
Cold Ischemia Time (min)	1.005 (1.003–1.008)	**<0.001**	–	–
Total Operation Time (min)	1.623 (1.323–2.058)	**<0.001**	1.517 (1.157–2.022)	**0.002**
Anhepatic Phase (min)	1.018 (1.006–1.031)	**0.004**	1.013 (0.997–1.027)	0.07
Portal Perfusion to Arterial Anastomosis Time (min)	1.007 (0.997–1.017)	0.116	–	–
Blood Loss (mL)	1.000 (1.000–1.001)	**0.014**	1.000 (1.000–1.001)	0.109
GRWR	0.930 (0.621–1.359)	0.718	–	–

**GRWR**: Graft-to-Recipient Weight Ratio.

### Multivariable Cox model for long-term GF risk (LASSO-selected)

Univariate Cox analysis (Tables 7–9) identified recipient PELD (HR = 1.031, *p* = 0.022), MELD (HR = 1.057, *p* = 0.010), platelet count (HR = 0.996, *p* = 0.039), albumin (HR = 0.890, *p* = 0.002), alkaline phosphatase (HR = 0.998, *p* = 0.012) and APRI–ALBI (HR = 1.045, *p* < 0.001); donor height (HR = 0.981, *p* = 0.010); and intra-operative cold ischaemia time (HR = 1.004, *p* < 0.001), total operative time (HR = 1.604, *p* < 0.001), portal-to-arterial anastomosis time (HR = 1.013, *p* = 0.005), blood loss (HR = 1.001, *p* < 0.001) and anhepatic phase (HR = 1.012, *p* < 0.001). After LASSO penalisation, the multivariable Cox model retained APRI–ALBI (HR = 1.027, *p* = 0.015), donor height (HR = 0.979, *p* = 0.012), total operative time (HR = 1.470, *p* < 0.001) and intra-operative blood loss (HR = 1.000 per mL, *p* = 0.002) as independent predictors of GF.

**Table 7.** Univariate and multivariate COX regression analysis of risk factors associated with graft dysfunction after liver transplantation in children with End-Stage liver disease.

Variable	Univariate analysis HR (95% CI)	P-value	Multivariate analysis HR (95% CI)	P-value
**Recipient Baseline Characteristics**				
Age (months)	1.007 (0.998–1.015)	0.13	–	–
Height (cm)	1.010 (0.995–1.025)	0.185	–	–
Weight (kg)	1.032 (0.994–1.072)	0.099	–	–
Recipient BMI (kg/m²)	1.011 (0.866–1.181)	0.888	–	–
**Disease Severity**				
PELD	1.031(1.004–1.058)	0.022	1.047 (0.913–1.202)	0.51
MELD	1.057(1.013–1.104)	0.01	0.974 (0.768–1.235)	0.826
Child-Pugh	0.997 (0.869–1.144)	0.965	–	–
**Preoperative Labs**				
PLT (×10⁹/L)	0.996 (0.992–1.000)	0.039	–	–
LYM (×10⁹/L)	0.887 (0.782–1.005)	0.060	–	–
CRP (mg/L)	1.018 (0.988–1.048)	0.244	–	–
TB (μmol/L)	1.002 (0.999–1.006)	0.213	–	–
DBIL (μmol/L)	1.002 (0.998–1.006)	0.395	–	–
ALB (g/L)	0.890 (0.828–0.957)	**0.002**	–	–
ALT (U/L)	1.000 (0.997–1.003)	0.899	–	–
AST (U/L)	1.001 (0.999–1.003)	0.383	–	–
Cr (μmol/L)	0.999 (0.986–1.012)	0.907	–	–
PT (s)	1.021 (0.959–1.086)	0.522	–	–
INR	1.172 (0.580–2.369)	0.658	–	–
AP (U/L)	0.998 (0.996–0.999)	**0.012**	1.000 (0.998–1.002)	0.954
CHE (U/L)	1.000 (1.000 − 1.000)	0.258	–	–
GGT (U/L)	0.998 (0.997–1.000)	0.065	–	–
APRI-ALBI	1.045 (1.027–1.063)	**<0.001**	1.027 (1.005–1.049)	**0.015**

**PLT:** Platelets; **LYM:** Lymphocytes; **ALT:** Alanine Aminotransferase; **AST:** Aspartate Aminotransferase; **ALP:** Alkaline Phosphatase; **GGT:** Gamma-Glutamyl Transferase; **TB:** Total Bilirubin; **DBIL:** Direct Bilirubin; **ALB:** Albumin; **CHE:** Cholinesterase; **Cr:** Creatinine; **PT:** Prothrombin Time; **INR:** International Normalized Ratio; **APRI:** AST to Platelet Ratio Index; **ALBI:** Albumin-Bilirubin; **PELD:** Pediatric End-Stage Liver Disease; **MELD:** Model for End-Stage Liver Disease.

**Table 8.** Donor baseline characteristics.

Variable	Univariate analysis HR (95% CI)	P-value	Multivariate analysis HR (95% CI)	P-value
Donor Age (years)	0.988 (0.938–1.041)	0.655	–	–
Donor Height (cm)	0.981 (0.966–0.995)	**0.01**	0.979 (0.963–0.995)	**0.012**
Donor Weight (kg)	0.979 (0.953–1.006)	0.13	–	–
Donor BMI (kg/m²)	0.931 (0.816–1.062)	0.287	–	–
Donor Age (years)	0.988 (0.938–1.041)	0.655	–	–

**Table 9.** Intraoperative parameters.

Variable	Univariate analysis HR (95% CI)	P-value	Multivariate analysis HR (95% CI)	P-value
Warm Ischemia Time (min)	1.098 (0.928–1.300)	0.276	–	–
Cold Ischemia Time (min)	1.004 (1.002–1.006)	**<0.001**	1.002 (0.999–1.005)	0.155
Total Operation Time (min)	1.604 (1.384–1.858)	**<0.001**	1.470 (1.209–1.787)	**<0.001**
Portal Perfusion to Arterial Anastomosis Time (min)	1.013 (1.004–1.022)	**0.005**	–	–
Blood Loss (mL)	1.001 (1.000–1.001)	**<0.001**	1.000 (1.000–1.001)	**0.002**
Anhepatic Phase (min)	1.012 (1.005–1.018)	**<0.001**	–	–
GRWR	0.949 (0.649–1.389)	0.788	–	–

**GRWR**: Graft-to-Recipient Weight Ratio.

### Nomogram construction and dose–response relationship

**Figure 1.**
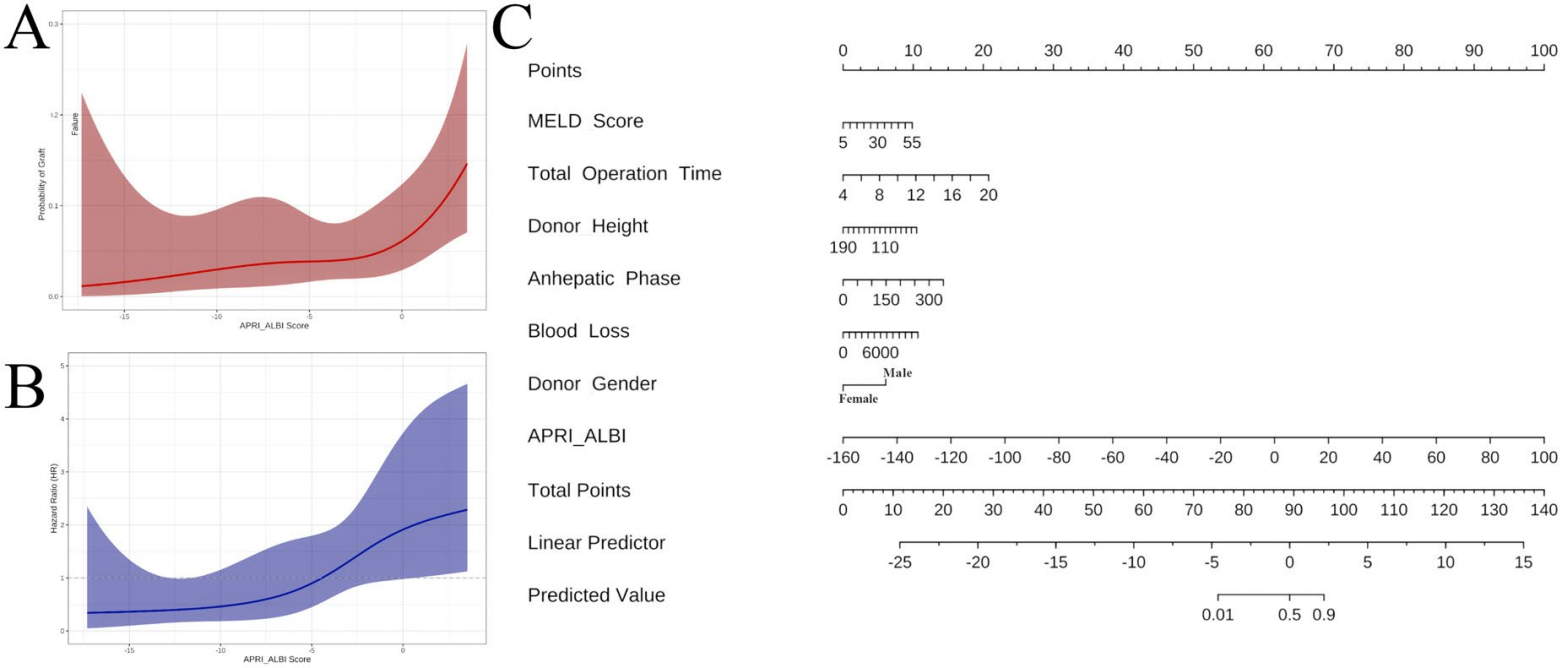
Predictive models for graft failure based on APRI_ALBI score. (a) The curve depicts the nonlinear association between APRI_ALBI score (x-axis) and predicted probability of graft failure (y-axis). Risk increases with rising APRI_ALBI, plateauing at scores above approximately 10. (b) The plot illustrates the continuous association between APRI_ALBI score (x-axis) and adjusted hazard ratio (HR, y-axis) for graft failure. Elevated APRI_ALBI scores correspond to progressively increased risk. (c) The nomogram integrates APRI_ALBI with seven clinical and donor-related predictors: MELD score, total operation time, donor height, anhepatic phase, blood loss, donor gender and APRI_ALBI. The total points correspond to a linear predictor and the predicted probability of graft failure, providing a clinically useful tool for individualized risk estimation.

A clinical nomogram (Figure 1(c)) integrating the seven independent predictors (MELD, donor sex, donor height, total operative time, anhepatic phase, blood loss and APRI–ALBI) was constructed to provide an individualised GF probability. Restricted cubic spline (RCS) logistic curves (Figure 1(a)) demonstrated a monotonic increase in short-term GF risk with APRI–ALBI, with a steep rise when the score exceeded 10. RCS Cox curves (Figure 1(b)) confirmed a parallel non-linear increase in long-term GF hazard.

### Model performance: discrimination, calibration and clinical utility

**Figure 2.**
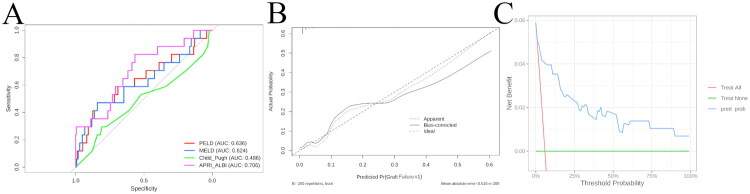
Performance evaluation and clinical utility of prediction models for graft failure. (a) Receiver operating characteristic (ROC) curves of four predictive models: PELD (AUC: 0.636), MELD (AUC: 0.624), Child–Pugh (AUC: 0.486), and APRI_ALBI (AUC: 0.706). (b) Calibration curve of the graft failure prediction model. The dashed line represents perfect calibration. The solid line indicates the model’s predicted probabilities against observed outcomes, with a mean absolute error of 0.018 based on 289 samples (B = 200 bootstrap repetitions). (c) Decision curve analysis (DCA) for the prediction model (pred_prob), compared with the strategies of treating all patients or treating none. The y-axis represents net benefit, and the x-axis indicates threshold probability.

ROC analysis (Figure 2(a)) showed the APRI–ALBI-inclusive model achieved an AUC of 0.706, outperforming PELD (0.636), MELD (0.624) and Child-Pugh (0.486); however, DeLong tests indicated no statistically significant pairwise differences (all *p* > 0.05). Bootstrap calibration (*B* = 200) revealed excellent agreement between predicted and observed probabilities, with a mean absolute error (MAE) of 0.018 (Figure 2(b)). Hosmer–Lemeshow tests for all models yielded *p* > 0.05, confirming adequate calibration. Decision-curve analysis (Figure 2(c)) demonstrated net clinical benefit of the nomogram across threshold probabilities of approximately 25–75% compared with ‘treat-all’ or ‘treat-none’ strategies.

### Long-term GF prediction: Kaplan–Meier and time-dependent ROC

**Figure 3.**
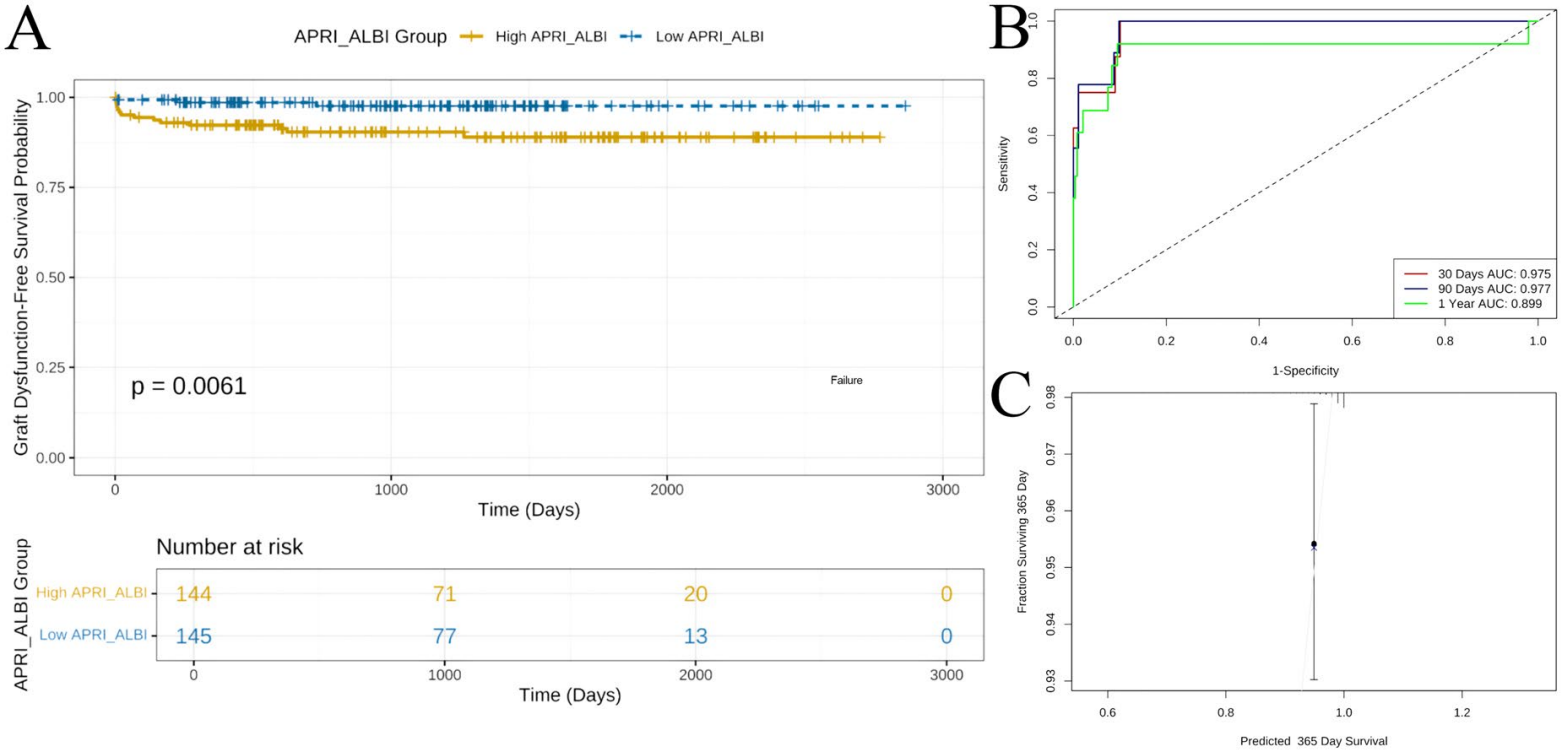
Survival analysis and time-dependent assessment. (a) Kaplan–Meier curves showed significantly lower graft failure-free survival in the high APRI-ALBI group (n = 144) than in the low-score group (n = 145; p = 0.0061); the separation persisted over 3,000 days, indicating that the score identifies recipients at early and sustained risk of graft failure. (b) Time-dependent ROC analyses of the Cox model achieved AUCs of 0.975, 0.977 and 0.899 at 30 days, 90 days and 1 year, respectively, demonstrating excellent short-term discrimination and maintained accuracy, validating APRI-ALBI for dynamic prognostication. (c) Decision curve analysis (DCA) for the prediction model (pred_prob), compared with the strategies of treating all patients or treating none. The y-axis represents net benefit, and the x-axis indicates threshold probability.

Kaplan–Meier analysis (Figure 3(a)) showed significantly lower GF-free survival in the high APRI–ALBI group (*p* = 0.0061); survival probabilities at 3 000 days were ≈ 0.20 vs 0.45 in the low-score group. Time-dependent ROC curves (Figure 3(b)) yielded AUCs of 0.975 (30 days), 0.977 (90 days) and 0.899 (1 year), indicating excellent short- and mid-term discrimination. One-year calibration (Figure 3(c)) confirmed close agreement between predicted and observed survival after optimism correction, underscoring the reliability of the model at the clinically relevant 365-day landmark.

## Post-operative complications and causes of graft failure

To further characterize the clinical profiles of the predicted risk groups, we analyzed post-operative vascular complications and the specific causes of graft failure. The overall incidence of hepatic artery thrombosis (HAT) and portal vein thrombosis (PVT) did not differ significantly between the High-Risk and Low-Risk groups (Supplementary Table S1; HAT: 4.8% vs. 3.5%, *p* = 0.563). However, the specific causes of graft loss revealed distinct patterns (Supplementary Table S2). Among the 17 patients with graft failure, those in the High-Risk group exclusively accounted for all fatal episodes of HAT (*n* = 1) and PVT (*n* = 1), as well as acute rejection (*n* = 2). In contrast, the rare graft losses in the Low-Risk group (*n* = 3) were driven solely by non-vascular factors, including primary non-function and severe biliary complications.

## Discussion

By retrospectively analysing 289 paediatric liver-transplant recipients, we systematically delineated the risk factors for GF and specifically evaluated the utility of the APRI–ALBI score for pre-operative risk stratification and outcome prediction. The principal findings are: (1) multiple recipient-, donor- and surgery-related variables are associated with an increased risk of GF; (2) multivariable modelling identified MELD score, donor sex, total operative time, APRI–ALBI score, donor height and intra-operative blood loss as key independent predictors; and (3) the resulting nomogram and APRI–ALBI-inclusive model demonstrated favourable performance and superior clinical net benefit compared with traditional scores.

First, univariate results are consistent with previous reports indicating that paediatric transplant outcomes are determined by a complex interplay of factors. On the recipient side, higher PELD/MELD scores, lower platelet and albumin levels, and an elevated APRI–ALBI – reflecting advanced fibrosis and impaired hepatic reserve – were all significantly associated with adverse outcomes [29,30]. These data underscore that baseline disease severity, synthetic function and underlying portal hypertension are critical determinants of prognosis. Male donor sex emerged as a risk factor, possibly related to hormonal-metabolic differences or subtle graft-quality disparities, although the exact mechanism remains speculative [31]. Intra-operative variables, including prolonged cold-ischaemia time, operative time, anhepatic phase and substantial blood loss, are well recognised to exacerbate ischaemia–reperfusion injury and surgical stress, thereby increasing the likelihood of GF [32].

An intriguing finding of our study was that children in the GF group were significantly older, presented with more severe preoperative illness, and underwent more complex surgical procedures compared to the non-GF group. This paradox can be explained by the specific disease characteristics within the GF cohort. Firstly, a considerable proportion of the GF group (41.2%) suffered from non-BA chronic conditions (such as Alagille syndrome, PBC, and cavernous transformation of the portal vein), which typically have a slower progression. These patients are often managed medically for years, resulting in an older age but a more debilitated systemic state at the time of decompensation. Secondly, among the BA patients in the GF group, several had undergone a prior Kasai portoenterostomy. While this procedure delays the necessity for transplantation – thereby increasing the age at surgery – it also causes dense intra-abdominal adhesions and exacerbates portal hypertension over time [33]. Consequently, these older patients face prolonged operative times, increased surgical bleeding, and highly challenging vascular reconstructions, all of which synergistically elevate the risk of early graft failure.

Second, LASSO regression and multivariable analyses refined these observations to a core set of independent predictors. In the logistic model, only MELD remained significant in the multivariable context, highlighting its established role in capturing transplant urgency and short-term risk. Conversely, in the Cox model – which better reflects temporal hazard – APRI–ALBI, donor height, total operative time and blood loss emerged as independent determinants of long-term GF. This divergence suggests that early GF is driven primarily by disease severity (MELD), whereas long-term dysfunction is modulated by chronic hepatic derangement (APRI–ALBI), graft quality/size mismatch (donor height as a surrogate for graft size) and cumulative surgical insult (operative time and haemorrhage). By integrating indices of portal hypertension (APRI) and hepatocellular reserve (ALBI), the APRI–ALBI score may more comprehensively capture the chronic pathological milieu influencing long-term graft performance [34].

Third, the APRI–ALBI-inclusive prediction model and nomogram exhibited favourable operating characteristics. Although the AUC of the composite model (0.706) numerically exceeded those of PELD, MELD and Child–Pugh, DeLong tests did not reach statistical significance – likely reflecting the small number of GF events (*n* = 17) and limited statistical power. Nevertheless, calibration curves and Hosmer–Lemeshow tests confirmed accurate alignment between predicted and observed probabilities. More importantly, decision-curve analysis demonstrated superior net clinical benefit across a wide range of threshold probabilities, supporting the practical value of the model for guiding clinical decisions such as intensified surveillance or pre-operative optimisation.

To further validate the clinical rationale of our risk stratification, we analyzed the specific mechanical causes of graft loss. Interestingly, the overall incidence of vascular complications like hepatic artery thrombosis (HAT) did not differ significantly between risk groups, likely because these events are strongly influenced by local anatomical and technical factors. However, the clinical consequences differed drastically. Among the rare graft failures in the predicted Low-Risk group (*n* = 3), none were attributed to vascular catastrophes like HAT; instead, they resulted from unpredictable events such as primary non-function or technical biliary complications. Conversely, graft losses directly caused by fatal HAT, PVT, or acute rejection occurred exclusively in the High-Risk group [35,36]. This indicates that our pre-operative predictive model successfully captures the systemic physiological exhaustion that makes recipients highly vulnerable to fatal post-operative vascular thromboses (a ‘failure to rescue’ phenomenon), while Low-Risk patients primarily succumb only to non-systemic, donor- or technique-related factors.

Of particular note, the APRI–ALBI-based Cox model excelled in sequential prediction. Time-dependent ROC curves revealed exceptional discrimination in the early post-transplant period (AUC > 0.975 at 30 and 90 days) that remained robust at one year (AUC = 0.899). Kaplan–Meier analysis corroborated these findings, showing significantly lower GF-free survival in children with high APRI–ALBI scores. Calibration plots further verified the accuracy of one-year survival predictions. Collectively, these data indicate that APRI–ALBI is not only a useful risk-stratification tool but also a powerful temporal prognostic indicator that can inform the intensity and timing of individualised post-operative follow-up.

Several limitations should be acknowledged. First, the retrospective, single-centre design may introduce selection bias, and the modest sample size – particularly the low event rate – could limit the stability and statistical power of multivariable analyses, possibly explaining the non-significant AUC comparisons. Second, although LASSO regression was employed for variable selection, potential confounders such as detailed immunosuppressive protocols and specific post-operative complications were not included. Finally, while internal bootstrap validation was performed, external validation in an independent, multi-centre cohort is required to confirm generalisability.

In summary, GF after paediatric liver transplantation results from a complex interaction of recipient, donor and surgical factors. The present study identified MELD score, APRI–ALBI score, donor characteristics (sex and height) and surgical parameters (operative time and blood loss) as key independent predictors. The APRI–ALBI-inclusive prediction model and nomogram demonstrated strong discrimination, calibration and clinical utility, especially for temporal outcome prediction. This provides clinicians with a visualisable risk-assessment tool to identify high-risk children pre-operatively, optimise donor–recipient matching, and tailor peri-operative management and individualised surveillance strategies, ultimately aiming to improve overall outcomes after paediatric liver transplantation. Prospective, multi-centre studies with larger event numbers are warranted to validate and refine these predictive models.

## Supplementary Material

Supplyment Table.docx

## Data Availability

The datasets generated and/or analysed during the current study are not publicly available due to patient privacy and ethical restrictions. However, a de-identified dataset may be made available from the corresponding author on reasonable request and with the approval of the Ethics Committee of the Children’s Hospital Affiliated to Chongqing Medical University.
