# Supplementary material for: Construction and validation of a prediction model for graft failure in pediatric liver transplant recipients based on the APRI-ALBI score: a retrospective study of 289 cases
Source: Ann Med. 2026 May 8;58(1):2654909. doi: 10.1080/07853890.2026.2654909 (PMC13159601; doi:10.1080/07853890.2026.2654909)
Supplement: Supplyment Table.docx [file IANN_A_2654909_SM6022.docx]

**Table S1. Incidence of post-operative vascular complications**

|  | **Total (n=289)** | **High-Risk Group(n=**145) | **Low-Risk Group(n=**144) | **P-value** |
| --- | --- | --- | --- | --- |
| Hepatic Artery Thrombosis (HAT), n (%) | 12 (4.1%) | 7 (4.8%) | 5 (3.5%) | 0.563 |
| Portal Vein Thrombosis (PVT), n (%) | 37 (12.8%) | 23 (15.9%) | 14 (9.7%) | 0.116 |
| Hepatic Vein / IVC Complications, n (%) | 16 (5.5%) | 8 (5.5%) | 8 (5.6%) | 0.981 |

**Table S2. Primary clinical causes of early graft failure among the 17 affected patients**

|  | **Total GF (n=17)** | **GF in High-Risk (n=14)** | **GF in Low-Risk (n=3)** |
| --- | --- | --- | --- |
| Hepatic Artery Thrombosis (HAT) | 1 | 1 | 0 |
| Portal Vein Thrombosis (PVT), n (%) | 1 | 1 | 0 |
| Primary Non-Function (PNF) | 4 | 3 | 1 |
| Severe Biliary Complications | 3 | 2 | 1 |
| Rejection | 2 | 2 | 0 |
| Other/Unknown Causes | 6 | 5 | 1 |
